# Short-Term Clinical Evaluation of Tibial Tunnel Angle and Position in Anatomical Anterior Cruciate Ligament Reconstruction

**DOI:** 10.3390/medicina61061107

**Published:** 2025-06-18

**Authors:** Mücahid Osman Yücel, Raşit Emin Dalaslan, Sönmez Sağlam, Mehmet Arıcan, Zekeriya Okan Karaduman, Bedrettin Akar

**Affiliations:** 1 Faculty of Medicine, Department of Orthopaedics and Traumatology, Duzce University, 81620 Duzce, Türkiye; 2 Department of Orthopedics and Traumatology, Sakarya Yenikent State Hospital, 54290 Sakarya, Türkiye

**Keywords:** anterior cruciate ligament, tibial tunnel, reconstruction, clinical outcomes, angle, position

## Abstract

*Background and Objectives*: This study aimed to evaluate the influence of the angle and position of the tibial tunnel in the coronal and sagittal planes on short-term postoperative clinical outcomes following arthroscopic anterior cruciate ligament reconstruction (ACLR). *Materials and Methods*: This retrospective study included 40 patients who underwent anatomical ACLR between 1 January 2023 and 31 December 2023 and had a follow-up period of at least 4 months. The angle of the tibial tunnel on the AP radiograph and both the angle and anteroposterior position on the lateral radiograph were measured. Clinical evaluations were conducted using the Visual Analogue Scale (VAS), the International Knee Documentation Committee (IKDC) score, and the Lysholm Knee Score, along with measurements of knee flexion and extension, to assess short-term outcomes at 1, 2, and 4 months postoperatively. *Results*: In patients whose tibial tunnels were positioned at 40–50° in the coronal plane, Lysholm scores were significantly higher at the 2nd and 4th months compared to other angles. In the sagittal plane, a tunnel angle between 30° and 40° was associated with significantly increased IKDC scores at both the 2nd and 4th months. Additionally, tunnels with an anterior–posterior ratio of 0.4–0.6 in the sagittal plane were associated with limitations in flexion and extension at the 4th month. There was no significant difference in VAS scores between the groups. *Conclusions*: Our findings suggest that optimizing the tibial tunnel angle in both the coronal and sagittal planes may play a crucial role in early postoperative knee function. Specifically, tibial tunnels placed between 40° and 50° in the coronal plane and 30° and 40° in the sagittal plane were associated with higher functional scores. However, tunnels positioned with an anterior–posterior ratio of 0.4 to 0.6 were linked to greater joint motion limitation. These findings indicate that angular and positional optimization of the tibial tunnel may have contributed to improved functional recovery following ACL reconstruction.

## 1. Introduction

Anterior cruciate ligament (ACL) injuries are a common orthopedic problem that affects a wide range of patients, particularly active athletes, and severely impairs the stability of the knee joint. The ACL plays a crucial role in maintaining the biomechanical integrity of the knee by limiting anterior tibial translation, controlling tibial rotation, and balancing valgus and varus stresses [1]. ACL tears lead to instability in the knee and increase the risk of additional pathologies such as meniscal and cartilage damage. This may lead to joint degeneration and long-term functional loss. Surgical reconstruction aims to accelerate the functional return of patients and maintain joint health by restoring knee stability [2].

On the other hand, the prognosis of ACL injuries treated with non-surgical methods is generally poor. These patients report lower satisfaction and functionality compared to those who undergo surgical reconstruction [3]. ACL injuries can significantly impact quality of life by disabling individuals from participating in sports activities and daily life [4]. Therefore, effective treatment of ACL injuries is crucial. It helps athletes maintain their careers and allows the general population to preserve functional independence [5].

Anterior cruciate ligament reconstruction (ACLR) is currently performed with various surgical techniques and is recognized as a procedure with high clinical success rates [6,7]. In both transtibial and anteromedial portal techniques, drilling the tibial tunnel at the correct angle is a key factor for surgical success [8,9,10,11,12,13,14,15,16,17,18]. The ideal tunnel angle and position enhance postoperative knee stability and function [19,20,21,22,23,24,25]. Previous studies have shown that the tibial tunnel angle can be measured with imaging methods such as X-ray and MRI [26]. Nonetheless, the optimal tibial tunnel angle and location remain a matter of controversy.

In this study, we evaluated the relationship between tibial tunnel angle and position, as well as postoperative functional outcomes in patients undergoing ACL reconstruction (ACLR). We analyzed the distribution in both the coronal and sagittal planes, comparing the clinical results of different angle and position groups. Our findings aimed to clarify surgical procedures by contributing to the determination of optimal tunnel angles and positions during ACL reconstruction (ACLR). Additionally, the results were intended to aid in predicting postoperative clinical outcomes, evaluating patients, and determining the rehabilitation process. We hypothesized that specific ranges of tibial tunnel angle and sagittal plane position would be associated with improved short-term functional outcomes after anterior cruciate ligament reconstruction (ACLR).

## 2. Materials and Methods

This study was retrospective in design. This study used data from patients who underwent ACLR at a university hospital between 1 January and 31 December 2023. The relevant ethics committee’s approval was obtained for this study, and all procedures were conducted following the Declaration of Helsinki.

**Figure 1:**
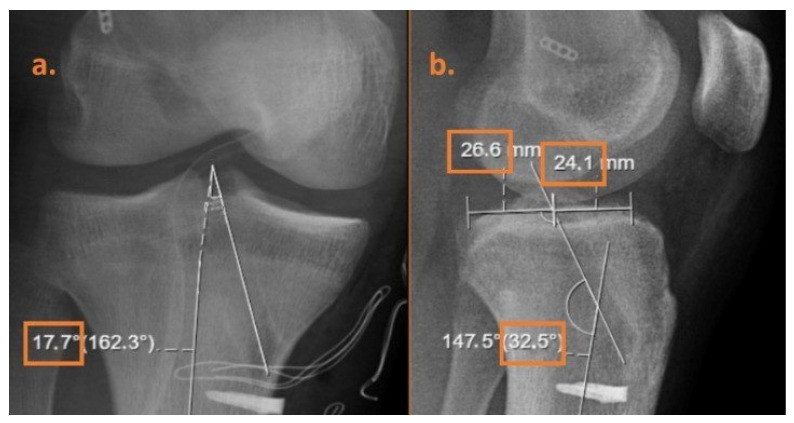
(**a**) The tibial tunnel angle was measured in the coronal plane as the angle between the tunnel axis and the anatomical axis of the tibia. (**b**) The tibial tunnel angle in the sagittal plane was measured, and the anterior–posterior position was calculated relative to the tibial plateau.

Inclusion criteria: Patients aged 15 to 50 years who underwent anatomical single-bundle ACLR, had complaints of knee instability, attended postoperative follow-up regularly, and had at least 4 months of clinical follow-up. Exclusion criteria: Patients with a history of previous knee surgery, intraoperative meniscal or cartilage lesions, or incomplete preoperative or postoperative clinical records. Patients were excluded if their follow-up visits differed by more than one week from the scheduled assessments at 1, 2, and 4 months, to maintain consistency in outcome evaluation. Clinical evaluations were performed with VAS (Visual Analogue Scale), IKDC (International Knee Documentation Committee) score, and Lysholm Knee Score. Postoperative anteroposterior (AP) and lateral knee X-ray images were obtained. Tunnel positioning was verified on postoperative radiographs to ensure anatomical placement. The tibial tunnel angle was measured as the angle between the tunnel axis and the anatomical axis in both sagittal and coronal planes (Figure 1). The tibial anatomical axis was defined as a line connecting the midpoints of medial–lateral cortices on AP and anterior–posterior cortices on lateral views. On lateral radiographs, distances from the tunnel exit point to the anterior and posterior tibial surfaces were measured separately (Figure 1). Then, the anterior/posterior distance ratio was calculated. Additionally, the angles of the tibial tunnel in the sagittal and coronal planes were analyzed in detail to assess graft placement and stability following surgery.

X-ray measurements were performed independently by two experienced orthopedic surgeons in a blinded fashion, and the agreement of the results was assessed. Interobserver reliability was evaluated by calculating the intraclass correlation coefficient (ICC) based on independent measurements from two observers. Radiographic measurements were performed using the hospital’s picture archiving and communication system (PACS). The X-ray measurements were compared with VAS, IKDC, and Lysholm scores, as well as knee flexion and extension range preoperatively and 1, 2, and 4 months postoperatively. In our study, the physical therapy protocol recommended by the Turkish Association of Orthopedics and Traumatology (TOTBID) was applied [27]. All patients underwent single-bundle ACLR with hamstring tendon autografts. The femoral tunnel was created via the anteromedial portal, and the graft was fixed with an adjustable loop.

Minor variations in femoral tunnel position were not considered exclusion criteria, as this study aimed to evaluate the effect of tibial tunnel angle on clinical outcomes. All femoral tunnels were placed according to standard anatomical landmarks, and no cases of clear malposition were observed on postoperative radiographs.

To enable meaningful subgroup comparisons, patients were stratified based on tibial tunnel angle and tunnel position values. Tunnel angles in the coronal and sagittal planes were grouped into 10° intervals (e.g., 30–40°, 40–50°), considering the natural distribution of the data and the need to maintain adequate sample sizes within subgroups. Similarly, tunnel positions in the sagittal plane were categorized according to anterior–posterior ratios (e.g., 0.4–0.6, 0.6–0.8). These grouping strategies were derived solely from the characteristics of the dataset. They were intended to ensure the statistical robustness of the analyses and facilitate the detection of potential differences across subgroups.

The aim was to determine whether a statistically significant relationship existed between the position and angle of the tibial tunnel and clinical outcomes. In this context, tibial tunnel angles and positions were assessed in the coronal and sagittal planes using AP and lateral knee radiographs, respectively. Patients were stratified according to angle and position groupings, and postoperative outcomes were evaluated using IKDC, Lysholm, and VAS scores, as well as knee flexion and extension measurements at 1, 2, and 4 months.

### Statistical Analysis

Descriptive statistics for continuous variables were presented as mean ± standard deviation, median, minimum, and maximum values. Percentages were used for categorical variables. The Shapiro–Wilk test was used to examine the conformity of continuous data to normal distribution. When comparing the scale scores between two groups of variables, the independent samples t-test was used for data that conformed to a normal distribution, and the Mann–Whitney U test was used for data that did not conform to a normal distribution. Kruskal–Wallis Analysis of Variance was used in comparisons of scale scores between variables with more than two groups. The Kruskal–Wallis Multiple Comparison test was used to analyze the groups from which the differences originated. The IBM SPSS for Windows 20.0 (SPSS Inc., Chicago, IL, USA) program was used in the evaluations, and *p* < 0.05 was accepted as the limit of statistical significance. Patients with missing data at any of the follow-up time points were excluded from the analysis. No correction for multiple comparisons was applied, as the analyses were exploratory. A priori power analysis was not performed due to the retrospective design and fixed sample size. However, post hoc effect sizes were evaluated where appropriate.

## 3. Results

The mean age of the patients was 26.8 years (range, 15–50), and the cohort included 38 males and 2 females. The mean BMI was 26.6 kg/m^2^ (range, 18.8–34.7). All patients completed at least 4 months of follow-up, and no graft failure, re-rupture, infection, or major complications occurred. All outcome measurements were obtained preoperatively and at 1, 2, and 4 months postoperatively. The interobserver agreement for tibial tunnel measurements was excellent, with an ICC of 0.89.

The most common coronal tibial tunnel angle was 30–40°, observed in 55% of patients. This was followed by the range of 40–50 degrees with 35%. Therefore, statistical comparisons were made between these two groups. In the sagittal plane, the most common angle range was found to be between 20 and 30 degrees with 30%. This was followed by 30–40 degrees with 27.5%, 10–20 degrees with 22.5%, and 40–50 degrees with 15%. This indicated greater variation in tunnel angles in the sagittal plane than in the coronal plane. According to the anterior–posterior ratio, 42.5% of the patients were in the range of 0.4–0.6. This group was followed by 0.6–0.8 in 30% and 0.8–1.0 in 17.5% (Table 1). In our study, the mean IKDC score was 51.58 ± 13.53, and the Lysholm score was 72.0 ± 17.90 at the 4th month evaluation of all patients.

There was a significant difference in Lysholm scores between patients with a tibial tunnel angle of 30–40° and 40–50° according to the coronal plane at 2 and 4 months postoperatively (*p* = 0.017, *p* = 0.035, respectively). Patients with 40–50° tunnel angles had higher Lysholm scores. However, no significant differences were found in IKDC, ROM, or VAS scores between the groups (*p* > 0.05) (Table 2).

There was a significant difference between the IKDC scores of patients with tunnel angles of 10–20, 20–30, 30–40, and 40–50 in the sagittal plane at 2 and 4 months (*p* = 0.004). The 30–40° group had significantly higher IKDC scores than the 10–20° and 20–30° groups at 2 months (*p* < 0.05, *p* < 0.01). No differences were found among the remaining groups. At 4 months, the IKDC scores of the patients with a 30–40° angle were significantly higher than the 10–20°, 20–30°, and 40–50° groups (*p* < 0.05). Apart from this, no significant difference was detected in terms of LYSHOLM, knee flexion, knee extension, and VAS scores preoperatively and at 1, 2, and 4 months postoperatively (*p* > 0.05). (Table 3).

At 4 months, significant differences in flexion and extension were observed between tunnel position groups (0.4–0.6, 0.6–0.8, 0.8–1.0) (*p* < 0.001). Knee flexion at 4 months was more limited in the 0.4–0.6 position group than in others (*p* < 0.001). Similarly, knee extension was also more restricted in the 0.4–0.6 group at 4 months (*p* < 0.001). There were no significant differences in IKDC, Lysholm, or VAS scores between the groups at any time point (*p* > 0.05) (Table 4).

In this study, we investigated the impact of the tibial tunnel angle and position on short-term functional outcomes in patients undergoing ACLR. Our findings showed that tibial tunnel angle and placement influenced some aspects of clinical recovery. Higher Lysholm scores were observed at 2 months (*p* = 0.017) and 4 months (*p* = 0.035) in patients with a coronal tibial tunnel angle between 40 and 50°. In the sagittal plane, IKDC scores were significantly higher in patients with tunnel angles of 30–40° at both the 2nd and 4th months (*p* = 0.004). Additionally, patients with tibial tunnel positions in the 0.4–0.6 anterior–posterior ratio range demonstrated more limited knee flexion and extension at 4 months (*p* < 0.001). These results suggest that tibial tunnel angle and position may play a role in optimizing short-term postoperative outcomes following ACLR.

## 4. Discussion

Descriptive statistics for continuous variables were presented as mean ± standard deviation, median, minimum, and maximum values. Percentages were used for categorical variables. The Shapiro–Wilk test was used to examine the conformity of continuous data to normal distribution. When comparing the scale scores between two groups of variables, the independent samples t-test was used for data that conformed to a normal distribution, and the Mann–Whitney U test was used for data that did not conform to a normal distribution. Kruskal–Wallis Analysis of Variance was used in comparisons of scale scores between variables with more than two groups. The Kruskal–Wallis Multiple Comparison test was used to analyze the groups from which the differences originated. The IBM SPSS for Windows 20.0 (SPSS Inc., Chicago, IL, USA) program was used in the evaluations, and *p* < 0.05 was accepted as the limit of statistical significance. * Patients with missing data at any of the follow-up time points were excluded from the analysis. No correction for multiple comparisons was applied, as the analyses were exploratory. A priori power analysis was not performed due to the retrospective design and fixed sample size. However, post hoc effect sizes were evaluated where appropriate.

Sim et al. reported no significant differences in tibial tunnel angles or IKDC scores between outside-in and transportal ACLR techniques [11]. Choi et al. found no significant differences in femoral tunnel angle or Lysholm and IKDC scores between anatomical and non-anatomical ACLR patients [9]. Similarly, Sundemo et al. reported that the femoral tunnel angle was not associated with the IKDC score in a study of patients undergoing non-anatomic ACLR [12]. However, studies in the literature have also shown that the coronal angle of the tibial tunnel does not affect IKDC scores [13,14].

Santhamoorthy et al. reported that maintaining a 41–51° coronal tibial tunnel angle in ACLR improved pivot shift and IKDC scores; however, they did not explain how this range was determined [15]. Ristic et al. also suggested that keeping the sagittal tibial tunnel angle in the range of 15–25° reduces the risk of complications [16]. Júnior et al. emphasized that a decrease in tibial tunnel coronal angle may improve IKDC and Lysholm scores [17]. A biomechanical study found that reducing the tibial tunnel’s coronal angle may increase PCL impingement and graft tension in flexion [18].

A coronal tibial tunnel angle of 40–50° was associated with better Lysholm scores at 2 and 4 months postoperatively (*p* = 0.017, *p* = 0.035). Tibial tunnels positioned at sagittal angles of 30–40° had significantly higher IKDC scores (*p* = 0.004). In our study, the IKDC score was 51.58 ± 13.53, and the Lysholm score was 72.0 ± 17.90 at 4 months. Ra et al. found that the IKDC score more sensitively reflects ACL-specific postoperative outcomes. They noted that the Lysholm score is less sensitive for ACL evaluation because it also includes meniscal pathologies [27]. The variation between coronal and sagittal plane scores suggests that changes in the coronal plane angle may be associated with increased meniscal stress. In addition, Ra et al. reported that the simpler and less detailed Lysholm score may result in higher scores than the IKDC [28]. This might have explained the higher Lysholm scores observed in our study. In addition, Nascimento et al. reported that IKDC scores rose from 55.3 preoperatively to 60.8 at six months, and Lysholm scores increased from 67.5 to 76.4 after ACLR [29]. Bodkin et al. reported that the IKDC score was 71 at 4 months and 83.47 at 6 months [30].

Howell et al. recommended that the coronal tibial tunnel angle should be kept at 15° or below. They reported that these values may lead to knee laxity by increasing joint motion limitation [8]. However, in our study, no significant relationship was found between tibial tunnel angle and range of motion.

In the literature, numerous studies have examined the impact of the tibial tunnel position in the sagittal plane on clinical outcomes, knee stability, and range of motion. For example, Inderhaug et al. showed that placing the tunnel more posterior than 1/1 increased rotational instability and negatively affected IKDC and Lysholm scores [14]. In contrast, Miller et al. used 2/3 as the reference point and found that anterior tunnel placement improved knee stability. However, IKDC scores and ROM were similar between anterior and posterior placements [20]. Behrend et al. also found that sagittal tunnel position had no significant effect on IKDC scores [13]. In our study, we hypothesized that the tunnel position in the sagittal plane would not have a substantial impact on IKDC and Lysholm scores (*p* > 0.05).

Ristic et al. reported that positioning the tunnel more anterior than 3/7 increased the graft failure rate [16]. Buyukdogan et al. reported that anterior placements above 2/3 offered better stability in pivot-shift tests but created a risk of excessive tension [21]. It is known that anterior placement increases the risk of notch impingement, and the limitation of extension may increase accordingly [22,23]. In general, the literature suggests that anterior positioning may result in loss of terminal extension, while posterior positioning may lead to flexion limitation [24,25]. Conversely, Romano et al. reported that the anterior positioning of the tibial tunnel may result in the loss of both extension and flexion [19]. Tibial tunnels positioned at 0.4–0.6 in the sagittal plane (i.e., more anterior) showed significantly greater limitations in knee extension and flexion at 4 months (*p* < 0.001). This suggests that the sagittal tunnel position has a minimal effect on early outcomes but may contribute to mid-term limitations in joint motion.

Additionally, Mann et al. found that the posterior position of the tibial tunnel increased the VAS score [31]. However, we did not observe a significant relationship between this parameter and the VAS score in our study.

Our study has several limitations. First, it employs a retrospective design and requires support from prospective and randomized controlled studies. Additionally, the absence of long-term follow-up data hindered our ability to fully demonstrate the effect of the tibial tunnel angle and position on long-term clinical outcomes. Multicenter studies with larger cohorts and extended follow-up will more clearly elucidate how an optimal tibial tunnel angle and position influence functional outcomes. In this retrospective study, we did not adjust for potential confounding factors, such as age, sex, BMI, and rehabilitation adherence, using multivariate analysis due to the limited sample size and the need for stratification into subgroups. Instead, we employed univariate statistical methods to explore associations. This limitation is acknowledged, and future research with larger cohorts should utilize regression models to better account for these variables.

## 5. Conclusions

Our findings suggest that the tibial tunnel angle and position may have influenced short-term clinical outcomes and range of motion following ACLR. Maintaining the tibial tunnel angle within the range of 40–50° in the coronal plane and 30–40° in the sagittal plane is associated with favorable improvements in clinical outcomes. Additionally, we considered that keeping the tibial tunnel position between 0.4 and 0.6 in the sagittal plane might have contributed to complications, such as joint motion limitation. These findings indicated that optimizing the angular and positional aspects of the tibial tunnel was critical for postoperative rehabilitation and functional success.

## Figures and Tables

**Table 1:** Distribution of tibial angles and positions.

Measured Tibial Tunnel Angle (°)	In Coronal Plane (*n*, %)	In Sagittal Plane (*n*, %)
10–20	-	9 (22.5%)
20–30	1 (2.5%)	12 (30.0%)
30–40	22 (55.0%)	11 (27.5%)
40–50	14 (35.0%)	6 (15.0%)
50–60	2 (5.0%)	2 (5.0%)
70–80	1 (2.5%)	-
**Tibial Tunnel Position**	**In Sagittal Plane (*n*, %)**	
0.4–0.6	17 (42.5%)	
0.6–0.8	12 (30.0%)	
0.8–1.0	7 (17.5%)	
1.0–1.2	3 (7.5%)	
1.2–1.4	1 (2.5%)	

*n*, number of patients; %, percentage of patients.

**Table 2:** Comparison of patients with tibial tunnel angles of 30–40° and 40–50° relative to the coronal plane.

Measured Tibial Tunnel Angle	In Coronal Plane 30–40° (*n* = 22)	In Coronal Plane 40–50° (*n* = 14)	
	Mean ± SD	Mean ± SD	*p* Value
IKDC (Preop)	43.14 ± 11.91	42.50 ± 13.29	0.882 ^a^
IKDC (Month 1)	43.32 ± 11.70	45.64 ± 12.60	0.576 ^a^
IKDC (Month 2)	48.09 ± 13.74	52.50 ± 12.01	0.332 ^a^
IKDC (Month 4)	48.82 ± 14.13	55.64 ± 13.34	0.158 ^a^
LYSHOLM (Preop)	64.14 ± 15.67	54.29 ± 21.02	0.117 ^a^
LYSHOLM (Month 1)	60.36 ± 16.31	70.86 ± 18.28	0.081 ^a^
LYSHOLM (Month 2)	66.95 ± 17.81	81.43 ± 15.32	**0.017 ^a^**
LYSHOLM (Month 4)	69.09 ± 16.66	81.07 ± 14.77	**0.035 ^a^**
Knee Flexion (Preop)	108.09 ± 28.27	112.36 ± 23.48	0.785 ^b^
Knee Flexion (Month 1)	87.95 ± 19.12	88.43 ± 20.39	0.936 ^b^
Knee Flexion (Month 2)	113.91 ± 12.86	113.93 ± 13.47	0.911 ^b^
Knee Flexion (Month 4)	118.00 ± 11.73	116.29 ± 9.15	0.343 ^b^
Knee Extension (Preop)	2.59 ± 3.81	2.36 ± 2.46	0.835 ^b^
Knee Extension (Month 1)	2.55 ± 3.23	1.86 ± 1.46	0.810 ^b^
Knee Extension (Month 2)	1.14 ± 1.24	1.07 ± 2.12	0.395 ^b^
Knee Extension (Month 4)	0.82 ± 1.18	1.00 ± 1.46	0.761 ^b^
VAS (Preop)	1.50 ± 2.17	2.21 ± 2.00	0.227 ^b^
VAS (Month 1)	1.45 ± 2.24	0.93 ± 1.14	0.885 ^b^
VAS (Month 2)	1.27 ± 2.05	0.71 ± 0.99	0.689 ^b^
VAS (Month 4)	0.86 ± 1.49	0.57 ± 1.08	0.575 ^b^

IKDC, International Knee Documentation Committee; LYSHOLM, Lysholm Knee Score; VAS, Visual Analogue Scale; preop, preoperative; SD, standard deviation; ^a^, independent samples *t* test; ^b^, Mann–Whitney U test.

**Table 3:** Comparison of patients with a tibial tunnel angle of 10–20°, 20–30°, 30–40°, and 40–50° relative to the sagittal plane.

	Measured Tibial Tunnel Angle in Sagittal Plane				
	10–20° (*n* = 9)	20–30° (*n* = 12)	30–40° (*n* = 11)	40–50° (*n* = 6)	
	Mean ± SD	Mean ± SD	Mean ± SD	Mean ± SD	*p* Value
IKDC (Preop)	40.00 ± 11.80	39.83 ± 12.77	49.00 ± 13.36	48.00 ± 9.61	0.197 ^d^
IKDC (Month 1)	42.44 ± 8.51	39.58 ± 12.08	50.64 ± 13.12	46.00 ± 10.23	0.199 ^d^
IKDC (Month 2)	45.67 ± 9.51	43.83 ± 10.96	62.88 ± 11.29	45.33 ± 7.68	**0.004 ^d^**
IKDC (Month 4)	46.33 ± 8.98	45.92 ± 11.45	65.73 ± 13.13	45.83 ± 8.70	**0.004 ^d^**
LYSHOLM (Preop)	60.33 ± 17.79	63.50 ± 21.58	60.82 ± 16.67	51.83 ± 18.25	0.603 ^d^
LYSHOLM (Month 1)	63.89 ± 24.35	63.92 ± 15.68	62.73 ± 16.34	67.83 ± 26.60	0.833 ^d^
LYSHOLM (Month 2)	69.56 ± 26.24	69.92 ± 18.28	74.82 ± 17.34	73.83 ± 17.94	0.933 ^d^
LYSHOLM (Month 4)	62.11 ± 20.48	75.42 ± 17.49	76.00 ± 16.22	74.83 ± 19.55	0.398 ^d^
Knee Flexion (Preop)	107.56 ± 27.54	96.25 ± 33.17	123.18 ± 10.78	113.83 ± 16.19	0.109 ^d^
Knee Flexion (Month 1)	86.67 ± 17.13	84.58 ± 23.97	93.91 ± 18.71	89.17 ± 9.70	0.837 ^d^
Knee Flexion (Month 2)	111.67 ± 12.03	111.25 ± 13.49	113.91 ± 14.59	121.33 ± 7.78	0.343 ^d^
Knee Flexion (Month 4)	119.78 ± 10.39	115.25 ± 13.63	117.55 ± 9.55	127.17 ± 6.49	0.673 ^d^
Knee Extension (Preop)	3.67 ± 4.87	3.08 ± 3.17	1.27 ± 1.73	0.50 ± 0.83	0.115 ^d^
Knee Extension (Month 1)	3.33 ± 3.04	2.92 ± 4.10	1.45 ± 1.36	1.67 ± 0.81	0.460 ^d^
Knee Extension (Month 2)	1.33 ± 1.58	0.83 ± 1.40	1.27 ± 2.45	1.00 ± 0.63	0.533 ^d^
Knee Extension (Month 4)	0.89 ± 0.92	1.00 ± 0.34	1.27 ± 11.55	0 (0–0)	0.118 ^d^
VAS (Preop)	1.89 ± 1.83	1.67 ± 2.18	1.18 ± 1.77	2.67 ± 2.80	0.594 ^d^
VAS (Month 1)	1.78 ± 2.58	1.25 ± 1.48	0.91 ± 1.92	1.50 ± 1.04	0.380 ^d^
VAS (Month 2)	1.00 ± 1.00	1.42 ± 2.23	0.73 ± 1.84	0.83 ± 0.98	0.546 ^d^
VAS (Month 4)	1.11 ± 1.45	0.50 ± 0.90	0.64 ± 1.80	0.67 ± 0.81	0.545 ^d^

IKDC, International Knee Documentation Committee; LYSHOLM, Lysholm Knee Score; VAS, Visual Analogue Scale; preop, preoperative; SD, standard deviation; ^d^, Kruskal–Wallis Analysis of Variance.

**Table 4:** Comparisons of patients with tunnel positions of 0.4–0.6, 0.6–0.8, and 0.8–1.0 according to the sagittal plane.

	Tibial Tunnel Position Relative to the Sagittal Plane			
	0.4–0.6 (*n* = 17)	0.6–0.8 (*n* = 12)	0.8–1.0 (*n* = 7)	
	Mean ± SD	Mean ± SD	Mean ± SD	*p* Value
IKDC (Preop)	42.82 ± 12.07	44.42 ± 12.13	47.86 ± 17.91	0.703 ^d^
IKDC (Month 1)	42.94 ± 15.20	43.08 ± 6.50	48.29 ± 9.08	0.470 ^d^
IKDC (Month 2)	48.71 ± 13.85	51.42 ± 10.29	47.57 ± 14.38	0.668 ^d^
IKDC (Month 4)	50.18 ± 14.38	53.67 ± 12.95	54.29 ± 17.15	0.706 ^d^
LYSHOLM (Preop)	62.29 ± 18.46	55.58 ± 18.72	65.57 ± 18.87	0.409 ^d^
LYSHOLM (Month 1)	64.12 ± 14.23	57.67 ± 21.69	62.14 ± 24.23	0.770 ^d^
LYSHOLM (Month 2)	68.53 ± 16.34	69.58 ± 22.30	73.14 ± 27.60	0.549 ^d^
LYSHOLM (Month 4)	70.12 ± 16.54	70.67 ± 18.30	76.14 ± 25.59	0.568 ^d^
Knee Flexion (Preop)	104.59 ± 30.49	114.58 ± 15.44	114.29 ± 18.12	0.573 ^d^
Knee Flexion (Month 1)	85.00 ± 22.50	89.83 ± 19.05	92.86 ± 3.93	0.828 ^d^
Knee Flexion (Month 2)	111.00 ± 13.46	109.17 ± 13.28	119.71 ± 10.90	0.126 ^d^
Knee Flexion (Month 4)	111.18 ± 9.82	118.75 ± 8.82	127.71 ± 3.54	**<0.001 ^d^**
Knee Extension (Preop)	2.06 ± 3.17	2.08 ± 1.83	1.57 ± 1.51	0.830 ^d^
Knee Extension (Month 1)	2.47 ± 3.48	1.92 ± 1.67	3.00 ± 3.55	0.946 ^d^
Knee Extension (Month 2)	1.06 ± 1.14	1.42 ± 2.35	0.86 ± 1.86	0.555 ^d^
Knee Extension (Month 4)	1.41 ± 1.06	0.42 ± 1.44	0.14 ± 0.37	**<0.001 ^d^**
VAS (Preop)	1.65 ± 2.23	2.08 ± 2.31	0.57 ± 0.97	0.360 ^d^
VAS (Month 1)	0.65 ± 0.86	0.75 ± 1.13	1.29 ± 1.49	0.594 ^d^
VAS (Month 2)	0.94 ± 1.91	0.92 ± 1.16	0.29 ± 0.48	0.624 ^d^
VAS (Month 4)	0.24 ± 0.56	0.58 ± 0.90	0.14 ± 0.37	0.272 ^d^

IKDC, International Knee Documentation Committee; LYSHOLM, Lysholm Knee Score; VAS; Visual Analogue Scale; preop, preoperative; SD, standard deviation; ^d^, Kruskal–Wallis Analysis of Variance.

## Data Availability

The dataset generated and analyzed during the current study is publicly available in the Zenodo repository at https://doi.org/10.5281/zenodo.15250515. This DOI represents all versions and will always resolve to the latest one.

## Abbreviations

The following abbreviations are used in this manuscript:

ACL	Anterior Cruciate Ligament
ACLR	Anterior Cruciate Ligament Reconstruction
AM	Anteromedial
AP	Anteroposterior
IKDC	International Knee Documentation Committee
Lysholm	Lysholm Knee Score
VAS	Visual Analogue Scale
TOTBID	Türk Ortopedi ve Travmatoloji Birliği Derneği
SD	Standard Deviation
